# Integration Profile and Safety of an Adenovirus Hybrid-Vector Utilizing Hyperactive Sleeping Beauty Transposase for Somatic Integration

**DOI:** 10.1371/journal.pone.0075344

**Published:** 2013-10-04

**Authors:** Wenli Zhang, Martin Muck-Hausl, Jichang Wang, Chuanbo Sun, Maren Gebbing, Csaba Miskey, Zoltan Ivics, Zsuzsanna Izsvak, Anja Ehrhardt

**Affiliations:** 1 Max von Pettenkofer-Institute, Department of Virology, Ludwig-Maximilians-University Munich, Munich, Germany; 2 Northwest Agriculture and Forestry University, Yangling, China; 3 Max Delbrück Center for Molecular Medicine, Berlin, Germany; 4 Ruhr-University of Bochum, Bochum, Germany; 5 Paul-Ehrlich-Institute, Division of Medical Biotechnology, Langen, Germany; 6 Institute for Virology and Microbiology, Center for Biomedical Education and Research, Department of Human Medicine, Faculty of Health, University Witten/Herdecke, Witten, Germany; Justus-Liebig-University Giessen, Germany

## Abstract

We recently developed adenovirus/transposase hybrid-vectors utilizing the previously described hyperactive Sleeping Beauty (SB) transposase HSB5 for somatic integration and we could show stabilized transgene expression in mice and a canine model for hemophilia B. However, the safety profile of these hybrid-vectors with respect to vector dose and genotoxicity remains to be investigated. Herein, we evaluated this hybrid-vector system in C57Bl/6 mice with escalating vector dose settings. We found that in all mice which received the hyperactive SB transposase, transgene expression levels were stabilized in a dose-dependent manner and that the highest vector dose was accompanied by fatalities in mice. To analyze potential genotoxic side-effects due to somatic integration into host chromosomes, we performed a genome-wide integration site analysis using linker-mediated PCR (LM-PCR) and linear amplification-mediated PCR (LAM-PCR). Analysis of genomic DNA samples obtained from HSB5 treated female and male mice revealed a total of 1327 unique transposition events. Overall the chromosomal distribution pattern was close-to-random and we observed a random integration profile with respect to integration into gene and non-gene areas. Notably, when using the LM-PCR protocol, 27 extra-chromosomal integration events were identified, most likely caused by transposon excision and subsequent transposition into the delivered adenoviral vector genome. In total, this study provides a careful evaluation of the safety profile of adenovirus/Sleeping Beauty transposase hybrid-vectors. The obtained information will be useful when designing future preclinical studies utilizing hybrid-vectors in small and large animal models.

## Introduction

As one of the best studied and the most utilized vectors in gene therapy, recombinant adenoviral vectors (AdVs) have the ability to mediate high efficient transduction in a broad range of cell types and tissues [1], [2]. The advanced version of AdVs represented by high-capacity adenoviral vectors (HC-AdVs) is characterized by the large cloning capacity of up to 36 kb due to the deletion of all viral coding sequences. Furthermore, HC-AdVs display a low toxicity profile and result in long-term transgene expression in quiescent cells. Therefore, AdVs have been applied in several inherited and acquired human diseases such as hemophilia, atherosclerosis, anemia, cystic fibrosis (CF) and Duchenne muscular dystrophy (DMD) with promising therapeutic effects [3]–[7].

However, as adenoviral vector genomes naturally remain episomally and integrate into host chromosomes at low frequencies [8]–[10], segregation of vector genomes to daughter cells during cell division is inefficient compared to integrating vector systems such as lentiviral vectors. As a result, adenoviral vectors are not applicable in gene therapy approaches aiming at stably transducing target tissues and cells with a high proliferative potential.

To prolong the therapeutic effect after adenoviral gene transfer in cycling cells and to stabilize the persistence of the therapeutic DNA at a lower dose, one promising approach is to combine HC-AdV with tools for somatic integration. A variety of adenoviral hybrid-vectors that lead to somatic integration of the transgene from the episomal adenoviral vector genome have been developed. These vectors combine the highly efficient DNA delivery of adenoviral vectors with integrating machineries for somatic integration of the therapeutic DNA, such as transposons (retrotransposon or Sleeping Beauty transposon) [11], [12], phage integrases (PhiC31) [13], retroviral/lentiviral integrases [14], or adeno-associated virus (AAV) derived Rep proteins [15], [16].

The SB transposase was reconstructed from a fish genome and belongs to the TC1/*mariner* family of transposable elements [17]. The SB recombination process is based on a cut-and-paste mechanism and results in integration of the transposon into a TA-dinucleotide of the host genome, which is the target site for the conventionally used version of SB. The original AdV/SB hybrid-vector system consisted of two independent HC-AdVs, of which one HC-AdV provided the integration machinery based on the unmodified version of the SB transposase and the other one contained the SB transposon carrying the transgene. This particular molecular design was called “two-vector-system” [12], [18]. For further improvements hyperactive versions of the SB transposase were generated including the hyperactive mutants HSB5 and SB100× with 10- to 100-fold increased integration efficiencies [19], [20]. Our recent study demonstrated that hyperactive transposase system (HSB5) delivered by high-capacity adenoviral vectors (HC-AdV) can result in somatic integration of a coagulation factor IX expression cassette in murine and canine liver, facilitating stabilized transgene expression and persistent hematological correction [21].

However, one important safety concern for all integrating vectors is represented by the risk of genotoxicity. Furthermore, exploration of potential dose effects of the AdV/SB hybrid-vectors is essential for evaluation of the pharmacokinetics and pharmacodynamics. Herein, we characterized sites of insertion after transposition from HC-AdVs *in vivo* by analysis of DNA derived from murine liver samples using linker-mediated PCR (LM-PCR) and linear amplification-mediated PCR (LAM-PCR). Integration site analysis revealed a close-to- random integration pattern with respect to integration into gene and non-gene areas and showed no significant differences regarding to the chromosomal integration. Furthermore, we found a correlation between the vector dose and the efficiency of the hybrid-vector system in terms of long-term transgene expression.

## Materials and Methods

### Sleeping Beauty Mediated Transposition in HeLa Cells

HeLa cells were maintained in Dulbecco’s modified Eagle’s medium (DMEM) (PAA) supplemented with penicillin/streptomycin (PAA), and 10% foetal bovine serum (FBS, Invitrogen). For transposition, HeLa cells were seeded into 6-well plates one day prior to the transfection. Cells at a confluency of 50–80% (SuperFect Transfection, QIAGEN) or 80% (FuGENE HD transfection, Promega) were co-transfected at a molar ratio of 3∶1 with 1.4 µg of the transposon donor plasmid pTnori [22] and various transposase encoding vectors. The latter vectors included the HSB5 encoding plasmids pCMV-HSB5 [20], the respective mutated and inactive SB (mSB) encoding plasmids pCMV-mSB [22] (for details regarding the plasmids please refer to **Figure S2a**). Forty-eight hrs post-transfection cells were split into 10-cm dishes at a density of 4×10^5^ cells and 4×10^4^ cells per 10-cm dish. After 14 days of constant selection pressure with 500 µg/ml G418, cells were either counted for the number of G418 resistant colonies or harvested as a transgene positive pool for recovery of integrated transposon sequences.

### Adenoviral Vector Production

**Figure 1:**
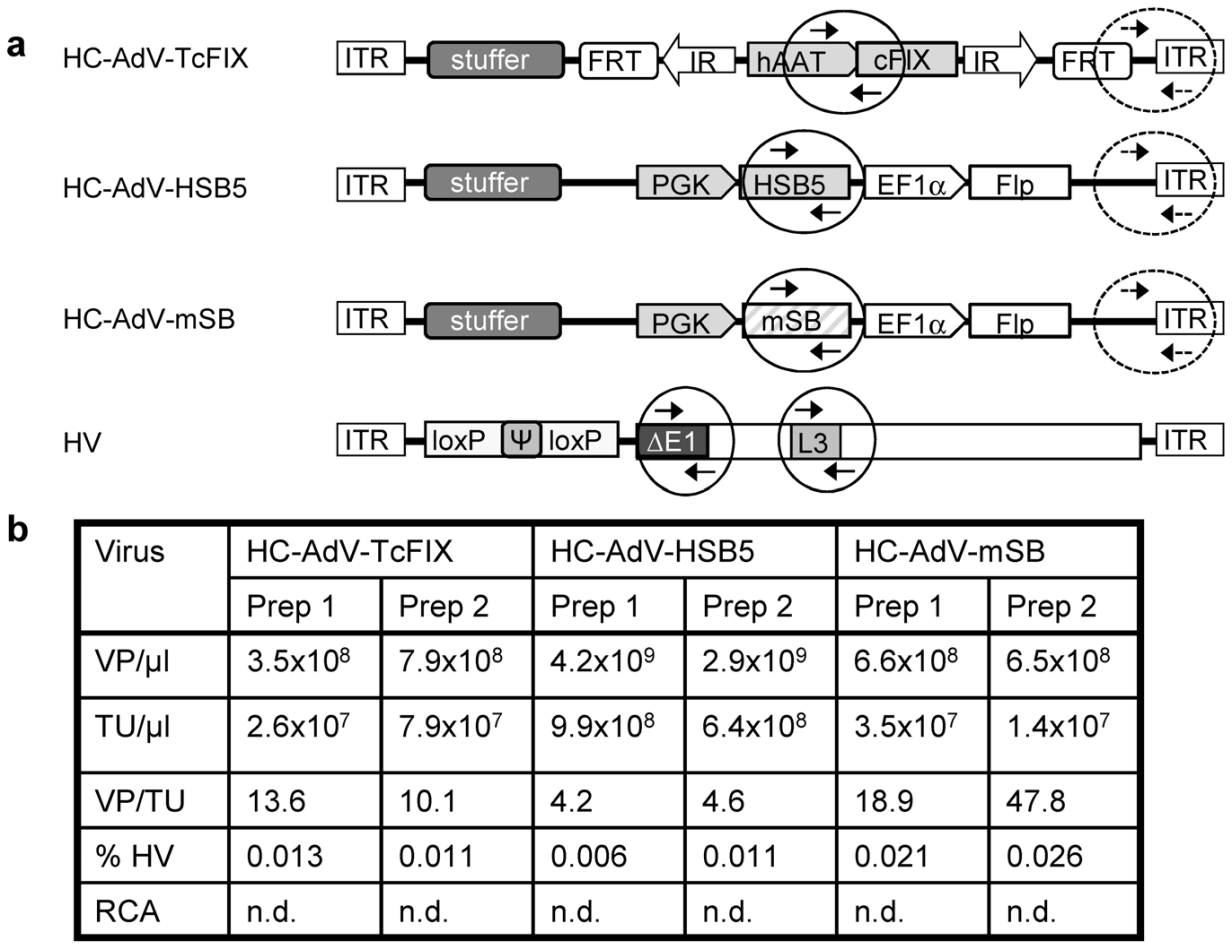
Characterization of the high-capacity adenoviral vectors (HC-AdVs) for *in vivo* applications. (**a**) Schematic outline of the viral vector constructs and location of real-time PCR primers for quantitative analysis of vector genome copy numbers. HC-AdV-TcFIX represents the transposon-donor vector for expression of canine coagulation factor IX under control of the liver specific human alpha-1-antitrypsin promoter (hAAT) including two liver specific enhancers (HCR: hepatocyte control region; ApoE: Apo lipoprotein E). Furthermore, the vector contains a transposon determined by inverted repeats (IRs) at the ends, which is flanked by two FRT sites for Flp-mediated excision. The HC-AdV-HSB5 contains a transgene expression cassette for the hyperactive Sleeping Beauty (SB) transposase (HSB5) under the control of the phospho-glycerate kinase promoter (PGK) and an expression cassette for the Flp recombinase driven by the elongation factor-1-alpha promoter (EF1α). The control vector HC-AdV-mSB contains the inactive version of the SB (mSB). All HC-AdVs include 22 kb stuffer DNA derived from human and mouse chromosomal DNA [3], [25]. Arrows inside the circles depict the real-time PCR primer binding sites specifically detecting vectors HC-AdV-TcFIX, HC-AdV-HSB5 and HC-AdV-mSB. To detect the HC-AdV backbone we performed a PCR detecting a region in close proximity to the right adenoviral ITR (marked with dashed circles). For evaluation of the HV contamination levels, a primer pair binding to the L3-encoded penton gene was used and to detect E1 back-recombination which may cause generation of replication competent adenovirus (RCA), primers specific to the E1 region were used. (**b**) Titer and purity of the final adenoviral vector preparations. The table summarizes the titration results and contamination levels with the helper virus used for vector production. Shown are six representative preparations of the high-capacity vectors which were used for hepatic infusion into C57Bl/6 mice. Physical titers were determined by OD_260 nm_ measurements. Titration using quantitative real-time PCR was carried out to measure infectious titers and HV contamination. The level of helper-virus contamination was defined as the percentage of transducing helper-virus particles contained in the total amount of transducing units of the respective HC-AdV. Abbreviations: VP, viral particle; TU, transducing unit; RCA, replication competent adenovirus; n.d., not detectable.

All viral vectors used in this study were described in our earlier study [21], [23] and produced according to an established protocol [24], [25]. All HC-AdVs include 22 kb stuffer DNA derived from human and mouse chromosomal DNA [3], [25] to optimize packaging of viral vectors. A detailed description of the principle of the adenovirus/transposase hybrid-vector system can be found in our previous publication [21]. The helper virus (HV) AdNG163R-2 used for the HC-AdV production is an E1-deleted first generation adenoviral vector [24]. For viral vector characterization and titration, quantitative real-time PCR (qPCR) with the primer pairs specific for the respective transgenes (HSB5/mSB and cFIX) as well as E1 and L3 genes were performed. For detailed description of each primer and titration results please refer to **Fig. 1** **and Table S1.** For titration of the HC-AdV-TcFIX vector, a 150 bp PCR product was amplified. For characterization of vectors HC-AdV-HSB5 and HC-AdV-mSB, the same primer set was used to amplify a specific 82 bp PCR product contained in the SB transposase gene. To detect the HC-AdV backbone we performed a PCR detecting a region in close proximity to the right adenoviral ITR. For evaluation of the HV contamination levels, a primer pair and a respective probe binding to the L3-encoded penton gene was used to amplify and detect a 67 bp PCR product. For detection of E1 back-recombination which may cause generation of replication competent adenovirus (RCA), primers and probe specific to the E1 region were used for amplification and detection of a 436 bp PCR product [26]. Genomic DNA isolated from viral vector infected 293-cells was used as templates for PCR reactions, except for E1-specific PCR which analyzed genomes isolated from purified viral particles.

### Animal Studies

This study was carried out in strict accordance with guidelines and regulations of the Government of Upper Bavaria in Germany. The protocol was approved by the Government of Upper Bavaria in Germany according to the animal protection law (TierSchG) (File number: 55.2-1-54-2531-92-06). All procedures were performed under isoflurane anesthesia and all efforts were made to minimize suffering. C57Bl/6 mice (6–8 weeks old) were kept and treated according to the guidelines of the Ludwig-Maximilians-University Munich. Animal experiments were usually ended when stabilized transgene expression levels were observed in mice which received the hyperactive SB transposase and when significantly decreased transgene expression were measured in the groups which received the inactive or no SB transposase. At the end of the experiment animals were sacrificed. Humane euthanasia was performed based on carbon dioxide and murine liver was removed post mortem. As summarized in **Table S2**, the highest viral vector dose was accompanied by cases of sudden death around one week post vector administration with, until now, unknown reasons. The condition of these mice was checked daily and the animals showed no signs of suffering or impending death. All mice were tail vein injected using a total volume of 200 µl. Viral vector preparations were diluted in Dulbeccós phosphate-buffered saline (DPBS). Rapid cell cycling of murine liver cells was induced by intraperitoneal administration of 25 µl carbon tetrachloride (CCl_4_, Sigma-Aldrich) diluted at a 1∶1 ratio with mineral oil.

### Blood Analysis

For the quantitative determination of alanine transaminase (ALT) in murine serum as signal for liver toxicity, the ALT kit (Randox) was used according to the manufacturer’s instructions. 10 µl of fresh murine serum were applied to each reaction. The measurement was conducted in the Ultrospec 3000 spectrophotometer (Pharmacia Biotech).

The cFIX enzyme-linked immunosorbent assay (ELISA) measuring cFIX levels in murine serum was performed as described earlier [21]. In brief, for coating of ELISA plates, the polyclonal rabbit anti-cFIX primary antibody (RACIX-IG, Affinity Biologicals Inc.) was diluted 1∶1000. After incubation with samples obtained from treated animals the sheep horseradish peroxidase–conjugated secondary antibody (SACIX-HRP, Affinity Biologicals Inc.) was added at a dilution of 1∶1000. For generation of a standard curve, dilutions of pooled dog plasma with a normal range of 5 to 11.5 µg/ml cFIX antigen were used.

### Generation of Sleeping Beauty Transposase-mediated Integration Libraries Using LM-PCR and LAM-PCR

We pursued a ligation-mediated PCR (LM-PCR) approach for isolation of transposition events. For construction of DNA libraries high-quality genomic DNA was prepared utilizing a protocol based on phenol-chloroform extraction. The isolation procedures for genomic DNA were described elsewhere [13], [25]. To ensure that the isolated genomic DNA is of adequate quality, it was checked on a 0.6% agarose gel visualizing non-fragmented chromosomal DNA. To generate SB integration libraries, 4 blunt-end digestions with *Ssp*I, *Pvu*II, *Eco*RV, or *Stu*I were performed by cutting 2.5 µg of high-quality genomic DNA with 80 units of the respective restriction enzyme in a total volume of 100 µl for each digestion. To ensure complete digestion, setups were incubated at 37°C for 16 to 18 hrs.

Before setting up the ligation reaction, the digestion reaction was purified by phenol-chloroform extraction. 100 µl phenol:chloroform:isoamyl alcohol (25∶24:1 vol/vol) were added and transferred into phase lock gel tube (PLG heavy, 5Prime Phase Lock Gel™) followed by centrifugation at high speed (15,000 *g*) for 5 min. Then, the nucleic-acid-containing aqueous phase was carefully transferred into a fresh tube, followed by ethanol precipitation. The pellet was dissolved in 20 µl of Tris-EDTA puffer (pH 7.5) for ligation. BD GenomeWalker™ Adaptors (Cat.No. 638901, Clontech, Heidelberg, Germany) were then ligated to both blunt-ends of the genomic DNA fragments to create libraries. The special amine in 3′end of the short strand insures the adaptor only ligated to blunt-end-genomic DNA fragments and only in one direction. A nested PCR utilizing adopter-specific primers (AP1 and AP2) and gene-specific primers (GSP1 and GSP2) was performed. GSPs should be derived from sequences as close to the end of the known sequence as possible. We wanted to analyze the integration sites after SB transposase-mediated integration and therefore, we designed primers which bind to the transposon-specific inverted repeat (IR) sequences. The two 27-mers primers, IR-specific primer 1 (GSP1, 5′-cct taa gac agg gaa tct tta ctc gga-3′) and IR-specific primer 2 (GSP2 5′-ggc taa ggt gta tgt aaa ctt ccg act -3′) were used for the two-step PCR.

For efficient amplification of genomic DNA templates of all sizes, the BD Advantage™ 2 Polymerase Mix (Cat.No. 639201, Clontech, Heidelberg, Germany) was used for PCR amplification. For the primary PCR we used the primers GSP1 and AP1 (5′- GTAATACGACTCACTATAGGGC -3′). We performed a two-step-PCR with 7 cycles at 94°C for 25 sec, 72°C for 3 min and 32 cycles at 94°C for 25 sec, 67°C for 3 min. After the final cycle, an extension was done with another 7 min at 67°C. Five µl of the primary PCR products were analyzed on a 1.5% agarose gel. Using 10-fold diluted primary PCR product as template, a secondary PCR with primers GSP2 and AP2 (5′- ACTATAGGGCACGCGTGGT -3′) was performed based on a two-step-PCR with 5 cycles at 94°C for 25 sec, 72°C for 3 min and 20 cycles at 94°C for 25 sec, 67°C for 3 min following with an extension for another 7 min at 67°C. Five µl of the secondary PCR products was analyzed on a 1.5% agarose gel. PCR products were subcloned using the Zero Blunt™ TOPO™ PCR Cloning Kit (Invitrogen) and pre-screened by EcoRI restricted enzyme digest flanking the multiple cloning site. Only plasmids which contained a unique DNA segment larger than 200 bp were sequenced utilizing the T7 and SP6 primers. For detailed description of this method and control steps see **Figures S2 and S3.** For the construction and identification of integration libraries from in vitro transposition in HeLa cells, a previously described plasmid rescued method was utilized [27]. The generation of Sleeping Beauty transposase-mediated integration site libraries via LAM-PCR in combination with an Illumina Genome Analyzer platform was performed as described previously [28]–[30]. Briefly, three enzymes *NlaI*II (NEB), *Mlu*CI (NEB), *Fsp*BI (Fermentas) were used to prepare independent reactions for LAM-PCR.

### Bioinformatic Analysis of Integration Sites

For LM-PCR, criteria and features of a transposition event are shown in **Figure. S3**. Obtained chromosomal sequences were blasted against the genomic sequence data base from NCBI, for human (http://www.ncbi.nlm.nih.gov/genome/seq/BlastGen/BlastGen.cgi?taxid=9606) and mouse (http://www.ncbi.nlm.nih.gov/genome/seq/BlastGen/BlastGen.cgi?taxid=10090). For LAM-PCR only the reads with exact adaptor sequence (23 nt) were retained for subsequent analysis. The remaining part of the single reads containing putative genomic sequences were cut after 50 nt. We mapped these reads to genome while only the sites with TA nucleotides were accepted. It is of note, that for the LAM-PCR dataset, extrachromosomal integration events referring to transposition events from the transposon-donor vector into the delivered episomal adenoviral vectors HC-AdV-TcFIX and HC-AdV-HSB5 were neglected. To map the individual integration events in mouse chromosomal DNA, the program Bowtie was used. To improve the data quality, we discarded all sequences for which we obtained <8 reads. To map these integrations identified by LAM-PCR back to individual chromosomal DNA, the Ensembl genome browser was used (the GRCm38.p1, Jan 2012 assembly of the mouse genome). For statistical analysis, we used random control sets with 10,000 sites each as described previously [28]–[30]. Cancer-relative-genes which were directly hit by integration events or near (±5 kb) the integration sites were checked in a Cancer Gene Expression Databases (CGED, http://lifesciencedb.jp/cged/) [31].

### Quantification of Vector Genome Copies by Taqman qPCR

For cFIX and SB gene copy number analysis, the same primer pairs as for vector titration were used. A primer pair and probe binding to the sequence near 3end ITR of FTC backbone was used to determin the copy number of entire vector gemome. To normalize the different samples, the same amount of genomic DNA was analyzed by real-time PCR mouse TBP (TATA box binding protein) was used for mouse samples and for dog samples B2M (Beta-2-microgloblin). For detailed description of each primer see **Table S1.**

## Results

### Dose-dependent Transgene Expression Levels and Stability of Transgene Expression Levels during Cell Division In Vivo

For systemic administration of high-capacity adenoviral vectors (HC-AdVs), the gene transfer efficacy, as well as the severity of the acute toxicity is vector dose dependent [32] and therefore, it is important to optimize the vector dose in a pre-clinical setting. We previously established the prototype of Sleeping Beauty (SB) transposase-adenovirus (AdV/SB) hybrid-vector system [21]. This vector system was based on one vector containing a transposon encoding canine coagulation factor IX (cFIX) under the control of a liver-specific promoter (HC-AdV-TcFIX) and a second vector (HC-AdV-HSB5) for expression of the hyperactive SB transposase HSB5 based integration machinery. As negative controls the third vector encoded a mutated and inactive version of SB transposase (HC-AdV-mSB), and the fourth vector (HC-AdV-Luc) expressed firefly luciferase representing an unrelated control vector has been described elsewhere [23]. Several independent viral preparations of each HC-AdV were produced and amplified following a standard protocol for large-scale production of HC-AdV in spinner flasks and subsequent purification by cesium chloride (CsCl) gradients using ultracentrifugation [24], [25]. To ensure high-quality viral vector productions, all viral vectors were characterized with respect to total physical titers and infectious titers (**Fig. 1b**). Furthermore, low levels (<0.1%) of contamination with helper virus (HV) were confirmed and generation of replication competent adenovirus (RCA) during the amplification procedure was excluded (**Fig. 1b**).

**Figure 2:**
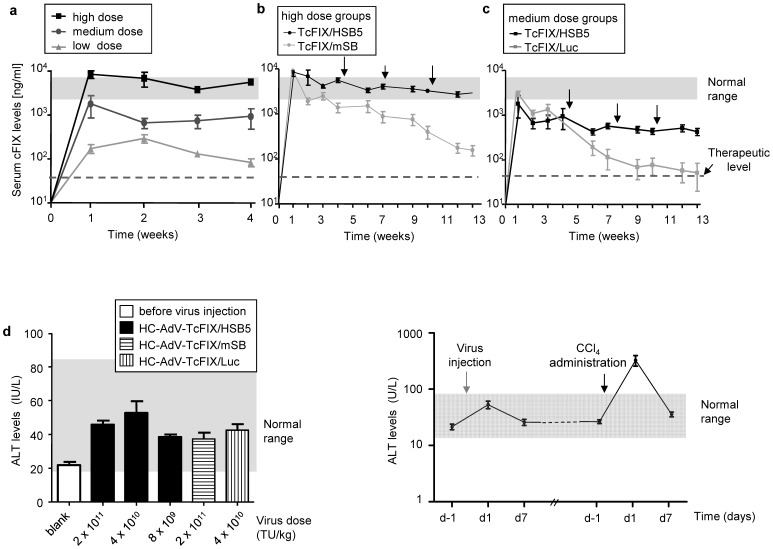
In vivo application of the AdV/SB hybrid-vectors in female mice. (**a**) Dose-dependent effects of the AdV/SB hybrid-vectors on transgene coagulation factor IX (cFIX) expression levels in female mice. Serum cFIX levels were measured weekly by ELISA during the first four weeks after vector injection and before CCl_4_ treatment for all three vector dose settings referred to as high (n = 3), medium (n = 5) and low dose (n = 5). A summary of vector injections and the viral load used is provided in Table 1. The normal range of cFIX (3000 to 8000 ng/ml) is indicated by a gray shadow. Notably, 50 ng/ml is sufficient for effective coagulation (therapeutic level, dashed line). Error bars indicate the standard deviations for each dose. (**b–c**) Stability of cFIX expression levels in actively dividing mouse hepatocytes. Mice were either co-injected with vectors HC-AdV-TcFIX and HC-AdV-HSB5 (TcFIX/HSB5) or HC-AdV-TcFIX and HC-AdV-mSB (TcFIX/mSB). Persistent transgene expression levels were observed in mice which received hyperactive transposase HSB5 (TcFIX/HSB5) in contrast to mice injected with the inactive SB (TcFIX/mSB) or an unrelated vector (TcFIX/Luc). Results are shown for mice treated with the high vector dose (2×10^11^ TU/kg; n = 3) (b) and the medium vector dose (4×10^10^ TU/kg; n = 5) (c). The normal range of cFIX (3000 to 8000 ng/ml) is indicated by as a gray bar. The therapeutic level (50 ng/ml) is shown as a dashed line. Arrows indicate the time points at that animals received intraperitoneal injections of CCl_4_ to promote hepatic cell cycling. Error bars indicate the standard deviations for each group. (**d**) Alanine transaminase (ALT) levels. ALT levels in serum samples of mice from different experimental groups were measured 1 day before and 1 day after viral vector injection (n = 5 per group, left panel). Long-term ALT levels were measured 7days post-vector injection and before and after CCl_4_ administration (right panel). The gray bar shows the normal range of ALT levels for mice (20–80 U/l). Error bars indicate the standard deviations for each group.

Herein, three different vector doses were chosen for systemic administration into female C57Bl/6 mice. In detail, low, medium and high total viral vector doses corresponding to 8.0×10^9^, 4×10^10^ and 2×10^11^ transducing units (TU) per kilogram body weight (kg), respectively, were injected into the tail vein. The ratio of co-injected vectors HC-AdV-TcFIX and HC-AdV-HSB5 used for the integrating hybrid-vector system was 3 to 1. As negative controls for somatic integration, mice of the control groups either received HC-AdV-mSB encoding an inactive version of SB or the vector HC-AdV-Luc as an unrelated control vector instead of HC-AdV-HSB5 at medium and high vector doses (4×10^10^ or 2×10^11^ TU/kg). A summary of all vector injections in mice is provided in **Table 1**. For mice which received the highest vector dose (2×10^11^ TU/kg), high levels of transgene expression were obtained (∼10 000 ng/ml cFIX measured by ELISA) **(** **Fig. 2a** **)**. However, this highest dose was accompanied by cases of death around one week post vector administration and a summary of treated mice and the respective survival rates are provided in **Table S2**. At the lowest dose, only low levels of serum cFIX were detected (∼100 ng/ml), but for the medium-dose group serum cFIX levels of up to ∼1000 ng/ml were measured over a time period of 4 weeks **(** **Fig. 2a** **).**

**Table 1:** Summary of vector injections in mice.

Dose/sex		Vector 1		Vector 2		Total	
	Weight	Type	TU/mouse	Type	TU/mouse	TU/mouse	TU/kg
**High-dose**							
Female	∼20 g	HC-AdV-TcFIX	3.0×10^9^	HC-AdV-HSB5	1.0×10^9^	4.0×10^9^	2.0×10^11^
Male	∼25 g	HC-AdV-TcFIX	3.75×10^9^	HC-AdV-HSB5	1.25×10^9^	5.0×10^9^	2.0×10^11^
Female	∼20 g	HC-AdV-TcFIX	3.0×10^9^	HC-AdV-mSB	1.0×10^9^	4.0×10^9^	2.0×10^11^
Male	∼25 g	HC-AdV-TcFIX	3.75×10^9^	HC-AdV-mSB	1.25×10^9^	5.0×10^9^	2.0×10^11^
Female	∼20 g	HC-AdV-TcFIX	3.0×10^9^	HC-AdV-Luc	1.0×10^9^	4.0×10^9^	2.0×10^11^
Male	∼25 g	HC-AdV-TcFIX	3.75×10^9^	HC-AdV-Luc	1.25×10^9^	5.0×10^9^	2.0×10^11^
**Medium-dose**							
Female	∼20 g	HC-AdV-TcFIX	6.0×10^8^	HC-AdV-HSB5	2.0×10^8^	8.0×10^8^	4.0×10^10^
Male	∼25 g	HC-AdV-TcFIX	7.5×10^8^	HC-AdV-HSB5	2.5×10^8^	1.0×10^9^	4.0×10^10^
Female	∼20 g	HC-AdV-TcFIX	6.0×10^8^	HC-AdV-Luc	2.0×10^8^	8.0×10^8^	4.0×10^10^
**Low-dose**							
Female	∼20 g	HC-AdV-TcFIX	1.2×10^8^	HC-AdV-HSB5	0.4×10^8^	1.6×10^8^	8.0×10^9^

Summarized are vector types and doses (high-dose, medium-dose and low-dose) of adenovirus/transposase hybrid-vectors and controls used for treatment of mice (n = 5 per group). In addition gender and body weight of treated mice are depicted. Abbreviations: HC-AdV, high-capacity adenoviral vector; TcFIX, the transposon encoding canine coagulation factor IX; HSB5, hyperactive Sleeping Beauty transposase; mSB, inactive Sleeping Beauty transposase; Luc, luciferase expression cassette; TU, transducing units.

To prove that SB-mediated integration from the episomal adenoviral genome can facilitate the stability of transgene expression levels *in vivo*, mice injected with hybrid-vectors and controls were treated with carbon tetrachloride (CCl_4_). CCl_4_ is a liver-toxic substance which causes death of affected hepatocytes by necrosis, and the amount of injected CCl_4_ (25 µl diluted in equal volume of mineral oil) has been shown to result in a destruction of about 70% of the whole liver [33]. During tissue repair rebuilding the liver to a normal size, hepatocytes are actively dividing and only transgenes stably integrated into chromosomal DNA are maintained. In contrast, episomal DNA without retention signals and origins of replication is not segregated to daughter cells during subsequent mitoses. As a result, the non-integrated molecular transposon forms will be lost after CCl_4_ treatment resulting in a decrease of transgene expression levels.

All experimental groups were treated 3 times with CCl_4_ beginning 4 weeks after vector administration with one injection every three weeks. During the time course of the experiment, serum cFIX expression levels were monitored by ELISA one day before CCl_4_ injection and at later time points once every week **(** **Figs. 2b, c** **)**. Mice which received the highest dose (2×10^11^ TU/kg) of the functional hybrid-vector system cFIX expression levels remained stable at physiological levels of cFIX (3000–5000 ng/ml) during the whole time course of the experiment. This was in sharp contrast to the control group which was treated with the same vector dose but with a vector expressing inactive SB (mSB). In this group cFIX levels showed a drop to 150 ng/ml after 13 weeks and three rounds of CCl_4_ treatment (**Fig. 2b**). For mice injected with the medium vector dose (4×10^10^ TU/kg) the same stability of transgene expression was observed after CCl_4_ treatment as demonstrated for the high vector dose group. Herein, stable cFIX levels (300–800 ng/ml) were measured throughout the whole experiment, whereas the control group receiving the same dose of the cFIX transposon donor vector HC-AdV-TcFIX but instead of the HSB5 encoding vector an unrelated vector (HC-AdV-Luc) serum cFIX expression levels decreased rapidly after induction of cell cycling. Although the cFIX levels (300–800 ng/ml) in the medium dose group were lower than the normal range of the physiological canine coagulation factor IX level (3000–8000 ng/ml), they remained above the therapeutic level for treatment of hemophilia B **(** **Fig. 2c** **).** However, for mice of the group which received the lowest vector dose (8×10^9^ TU/kg), low serum cFIX levels (∼100 ng/ml) were observed within the first few weeks dropping to undetectable levels at later time points after CCl_4_ administration (data not shown).

Limited toxicity studies were performed by monitoring alanine transaminase (ALT) levels in mouse serum samples of treated mice to detect acute toxicity associated with administration of HC-AdVs. For all study groups, ALT levels were measured one day before and one day post-injection. Although ALT levels were elevated compared to the levels before injection, levels remained in the normal range (20–80 IU/L) for all treated mice **(** **Fig. 2d,** left panel **)**. To conduct a long term survey of liver toxicity, two mice from each group were monitored and 7 days post-vector injection ALT levels decreased to similar levels as before vector injection **(** **Fig. 2d,** right panel **)**. To monitor indirectly hepatocytes’ damage, and to measure liver toxicity caused by CCl_4_ administration, ALT levels before and after CCl_4_ administration were measured in the same mice. Serum transaminase levels dramatically increased to up to 600 U/l at day 1 after CCl_4_ administration but declined to a normal range one week later **(** **Fig. 2d,** right panel).

### Integration Site Pattern Identified from Female Mice Treated with AdV/SB Hybrid-vectors Using LM-PCR and LAM-PCR

In previous clinical studies utilizing retroviral vectors for gene transfer and somatic integration genotoxicity was observed due to activation of oncogenes [34]–[36]. Thus it was of great importance to carefully investigate the risk of genotoxicity after SB-mediated transposition from the adenoviral vector. Therefore, after stable transgene expression was obtained in mice after systemic administration of the AdV/SB hybrid-vector system (**Figs. 2b, c**), integration sites were identified to evaluate the potential genotoxicity of the AdV/SB hybrid-vector system. We performed integration site analysis of genomic DNA derived from liver tissue samples of three treated female C57Bl/6 mice which showed stabilized and highest cFIX expression levels. In detail, genomic DNA was extracted from two high-dose HSB5-mouse after 9 weeks (f1) and after 13 weeks (f2), respectively. Furthermore, a liver sample of a mouse from the medium-dose HSB5-group 14 weeks post injection (f3) was analyzed. As control, genomic DNA sample from a mouse of the high-dose mSB-group 9 weeks after vector application (f4) was investigated. An overview of all analyzed mice for integration site analysis is provided in **Table 2**.

**Table 2:** Overview of mice used for integration sites analysis.

		Vector Injection		Parameters at the end of the study		
	Type of analysis	Type of vector	Dose (TU/mouse)	Time after injection (weeks)	cFIX level(ng/ml)	cFIX level of control group(ng/ml)
f1	LM-PCR/LAM-PCR	HC-AdV-TcFIX/HSB5	4.0×10^9^	9	4800*	750
f2	LM-PCR/LAM-PCR	HC-AdV-TcFIX/HSB5	4.0×10^9^	13	3100*	150
f3	LM-PCR	HC-AdV-TcFIX/HSB5	8.0×10^8^	14	520*	70
f4	LM-PCR	HC-AdV-TcFIX/mSB	4.0×10^9^	9	700*	NA
m1	LM-PCR/LAM-PCR	HC-AdV-TcFIX/HSB5	8.0×10^8^	14	510*	NC
m2	LM-PCR/LAM-PCR	HC-AdV-TcFIX/HSB5	8.0×10^8^	14	780*	NC

* Canine coagulation factor IX (cFIX) expression levels represent values obtained at the last measurement before extraction of liver genomic DNA.

Abbreviations: cFIX, canine coagulation factor IX; HSB5, hyperactive Sleeping Beauty transposase; mSB, inactive Sleeping Beauty transposase; TU, transducing units. f1: female mouse 1; f2: female mouse 2; f3: female mouse 3; m1: male mouse 1; m2: male mouse 2; f4 (mSB): mSB mouse (female mouse 4); LM-PCR, linker-mediated PCR; LAM-PCR linear amplification-mediated PCR; NA, not applicable; NC, not conducted.

Integration site analysis was performed utilizing a ligation-mediated PCR (LM-PCR) method (**Figure S1**) and a previously published linear amplification-mediated PCR (LAM-PCR) protocol [30], [37]. To establish the LM-PCR protocol for SB transposase mediated transposition, we first performed experiments in HeLa cells and evaluated the protocol at each step (**Figure S2**). Features of the expected transposition events using linker-mediated PCR (LM-PCR) are schematically shown in **Figure S3a** and examples for the HSB5 and mSB groups are shown in **Figure S3b**. **Figure S3c** shows an exemplary library derived from mouse f1 treated with cFIX and HSB5 expressing vectors and mouse f4, which received the inactive transposase (mSB) encoding vector. It is of note, that the number of clones containing unique fragments is significantly higher in mouse f1 than f4 treated with the inactive transposase (mSB) encoding vector.

**Figure 3:**
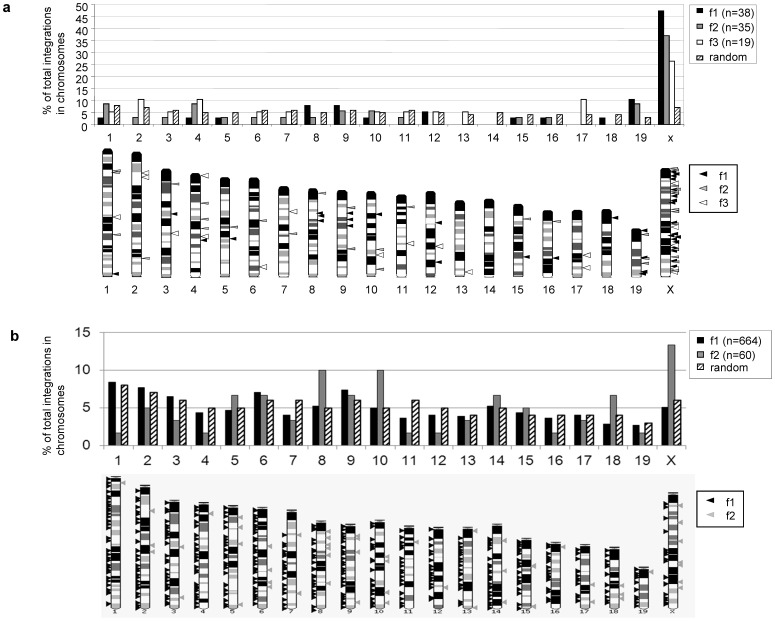
SB-mediated integration sites from the adenoviral vector in female C57Bl/6 mice using LM-PCR and LAM-PCR approaches. Chromosomal distribution of unique integration sites analyzed from female mice treated with functional AdV-SB hybrid vectors are compared to a computer-simulated random integration profile. (**a**) Integration events identified by LM-PCR. Percentages of integration events within each individual chromosome in comparison to the total number of identified integration sites are depicted on the upper panel. A schematic overview of chromosomal distribution is shown on the lower panel. Respective triangles indicate the relative positions of the chromosomal transposon integration site observed from liver genomic DNA of female mouse f1 (black triangles), female mouse f2 (grey triangles) and female mouse f3 (white triangles). (**b**) Sequence reads and chromosomal distribution identified after performing LAM-PCR from female mouse f1 and f2. Percentages of integration events within each individual chromosome in comparison to the total number of identified integration sites are depicted on the upper panel. The lower panel shows mapping of all 724 unique integration sites identified by LAM-PCR which were mapped within the mouse genome. Respective triangles indicate the relative positions of the chromosomal transposon integration site observed for liver genomic DNA of female mouse 1 (black triangles) and female mouse 2 (grey triangles).

Using the LM-PCR method a total of 92 unique SB-mediated integration sites (IS) were identified from female C57Bl/6 mice which received the high and medium dosages of the AdV/SB hybrid-vector system (38 IS from mouse f1; 35 IS from mouse f2; 19 IS from mouse f3). An overview of the integration site recovery rate of the LM-PCR is provided in **Table S3** and the chromosomal distribution of chromosomal integration events is shown in **Fig. 3a**. However, the limited number of recovered integration sites after performing LM-PCR motivated us to perform large-scale analysis of transposition events. Therefore, we applied a previously published LAM-PCR protocol using IR-specific primers [28]–[30] and obtained libraries and respective sequences were analyzed on an Illumina Genome Analyzer platform. Analysis of sequence reads on the mouse genome revealed a total of 724 unique integration sites in female mice f1 and f2 after transposition from the adenoviral vector with a close to random chromosomal integration profile (**Fig. 3b**).

### Stability of Transgene Expression Levels and Integration Site Profiling in Male Mice Treated with AdV/SB Hybrid-vectors

**Figure 4:**
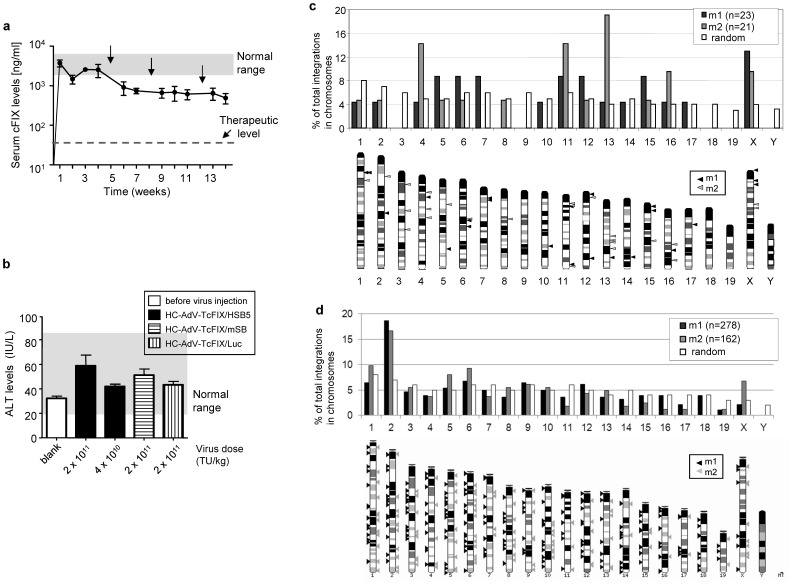
SB-mediated integration sites in male C57Bl/6 mice. (**a**) Stable cFIX expression in male mice after treatment with AdV/SB hybrid-vectors. Male C57Bl/6 mice (n = 5) were co-injected with HC-AdV-TcFIX and HC-AdV-HSB5 at a ratio of 3 to1 using a medium total vector dose (4×10^10^ transducing units (TU) per kilogram body weight [kg]). A detailed summary of vector injections and the viral load used is provided in **Table 1**. ELISA measurements for detection of canine factor IX in mouse serum were performed periodically and carbon tetrachloride (CCl_4_) injections are indicated as arrows. The normal range of cFIX (3000 to 8000 ng/ml) is indicated by gray bar. Notably, 50 ng/ml is sufficient for effective coagulation (therapeutic level). Error bars indicate the standard deviations for each group. (**b**) Alanine transaminase (ALT) levels. ALT levels in serum samples of male mice treated with functional AdV/SB hybrid-vectors (HC-AdV-TcFIX/HSB5; high dose: 2×10^11^ TU/kg; medium dose: 4×10^10^ TU/kg) as well as with non-functional variants (HC-AdV-TcFIX/mSB: high dose: 2×10^11^ TU/kg; HC-AdV-TcFIX/Luc: high dose: 2×10^11^ TU/kg) are shown. Samples from 1 day before and 1 day after vector injection were analyzed for all groups (n = 5 per group). The grey shadow shows the normal ALT range for mice (20–80 U/l). Error bars indicate the standard deviations for each group. (**c**) Chromosomal distribution of SB-mediated integration events in male C57Bl/6 mice using LM-PCR. Integration sites analyzed from genomic DNA derived from liver samples of two male mice (m1 and m2) treated with the AdV/SB hybrid vectors were compared to a computer-simulated random integration profile [23]. Percentages for integration events within each individual chromosome in comparison to the total number of identified integration sites are summarized (upper panel). The lower panel shows the overall distribution of transposition events in murine host chromosomes. Male mouse m1 (black triangles) and male mouse m2 (grey triangles) are shown. (**d**) Integration events identified from male mice after performing LAM-PCR. The relative position of all unique integration sites identified from male mice m1 and m2 after SB-mediated transposition from the adenoviral vector (AdV/SB hybrid-vector system) were identified by LAM-PCR. The top panel analyzes percentages of integration events within each individual chromosome in correlation to the total number of identified integration sites. The mapped integration sites are shown in the bottom panel. Respective triangles indicate the relative positions of the chromosomal transposon integration site observed for liver genomic DNA of male mouse m1 (black triangle) and male mouse m2 (grey triangles).

To rule out gender differences after transposition in mice which shown in a previous study [38], we investigated transgene expression levels and the chromosomal distribution pattern in male mice. Therefore, groups of male mice (n = 5) were injected with the AdV/SB hybrid-vector system (HC-AdV-TcFIX co-injected with HC-AdV-HSB5 at a ratio of 3∶1) applying a high dose (2×10^11^ TU/kg) or a medium dose (4×10^10^ TU/kg), respectively. As negative control for somatic integration, mice of the control group received HC-AdV-mSB encoding an inactive version of SB or the HC-AdV-Luc as an unrelated control vector at a high vector dose (2×10^11^ TU per mouse) (**Table 1**). Mice treated with a medium vector dose of HC-AdV-TcFIX/HSB5 showed stable transgene expression. Although serum cFIX levels decreased after the first CCl_4_ administration, they remained stable after the second and third CCl_4_ administration (**Fig. 4a**). However, as observed for female mice injected with high dose, also male mice of all three groups (HC-AdV-TcFIX/HSB5, HC-AdV-TcFIX/mSB and HC-AdV-TcFIX/Luc) died after application of the high vector dose. An overview of survival rates of treated mice is provided in **Table S2**. In concordance with ALT levels measured in female mice, slightly elevated ALT levels were measured 1 day post-injection for all treated male mice, while remaining within the normal range (20–80 IU/L) (**Fig. 4b**).

For generation of an integration site library, two male mice (m1 and m2) from the medium vector dose group with serum cFIX levels of 460 and 850 ng/ml were sacrificed 14 weeks post vector infusion (see also **Table 2**). As in female mice, for integration site analysis LM-PCR and LAM-PCR were performed. When applying the LM-PCR method 44 unique transposon insertions were identified in male mice (**Fig. 4c**). To obtain a larger number of integration sites we also performed LAM-PCR using liver samples from male mice m1 and m2 and analysis of sequence reads on the mouse genome revealed a total of 440 unique integration sites after transposition from the adenoviral vector (**Fig. 4d**). The chromosomal distribution of all transposition events revealed a close-to-random integration profile (**Fig. 4**).

### Frequencies of Transposon Insertions within or Outside of Genes and Transposition Events into Extra-chromosomal Target Sequences

The mutagenic potential of any given integrating vector system is one of the most essential considerations for gene therapy applications. Normally, the integrations in genes display a higher risk to cause genotoxicity than those which hit an intergenic region. Thus the relation of transposon insertions to transcriptional units was analyzed, aiming to determine the mutagenic potential associated with AdV/SB hybrid-vectors. To address this issue integration sites identified from the three female mice (f1, f2 and f3) and the two male mice (m1 and m2) were analyzed.

**Figure 5:**
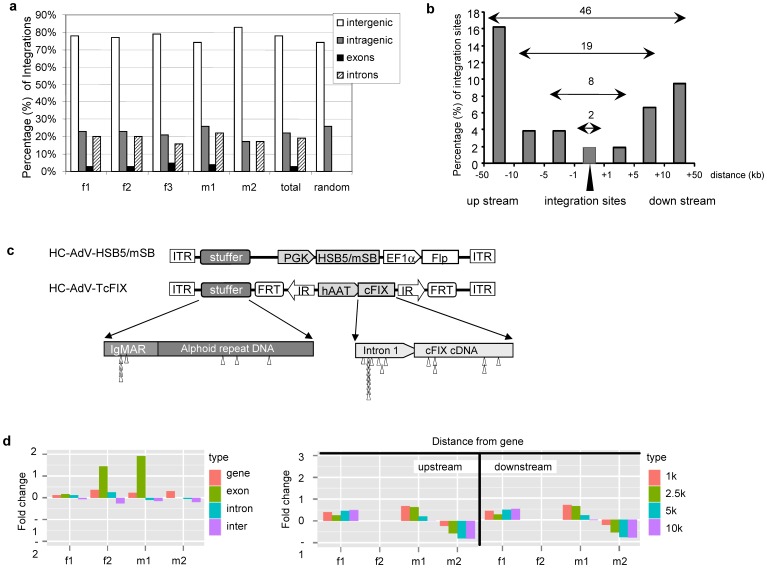
Frequencies of insertions rescued from murine liver within or outside of genes and extrachromosomal target sites after performing. (**a**) Percentage of integration sites which were identified to be intragenic, intergenic, in intron, or in exons after performing LM-PCR. f1: female mouse 1; f2: female mouse 2; f3: female mouse 3; m1: male mouse 1; m2: male mouse 2; total: all integration sites from these five mice; random: computer predicted data [53]. (**b**) LM-PCR derived integration sites and their proximity to genes. The number above the horizontal arrows indicates the number of integration events with varying proximities to genes. The X-axis describes the distance of each integration site upstream and downstream of the nearest gene in kilobases (kb). The Y-axis shows the percentage of integration events. (**c**) Transposition into extrachromosomal target sequences using the LM-PCR method. The upper panel schematically shows the vector constructs of the transposon encoding vector HC-AdV-HSB5/mSB and the transposon-donor vector HC-AdV-TcFIX. The lower panel shows a detailed map of the stuffer DNA and the transgene. The stuffer DNA consists of two parts: IgkMAR (the matrix attachment region of the murine immunoglobulin κ locus) and alphoid repeat DNA (alphoid repeat DNA sequences from human chromosome 17). The core transgene was represented by the canine coagulation factor IX cDNA (cFIX) including a portion of the first intron of the human FIX gene expressed under the control of the liver-specific alpha-1-antitrypsin promoter (hAAT). The triangles show the relative positions of the extrachromomal integrations sites which were mapped to the injected adenoviral vector genomes. IR, transposon derived inverted repeat; ITR, adenovirus derived inverted terminal repeat; PGK, phosphor-glycerate kinase promoter, FRT, FLP recombinase recognition sites; EF1α, elongation factor-1-alpha promoter; HSB5, hyperactive Sleeping Beauty transposase HSB5. (**d**) Characterization of adenovirus/transposase hybrid-vectors mediated integrations identified by LAM-PCR method. Insertion frequencies relative to a random dataset are shown with respect to integration within and outside RefSeq genes (left panel), and distance upstream and downstream of genes (right panel). The bars depict fold changes of integration frequencies relative to the random distribution profile.

When using the LM-PCR method, 31 (23%) of the 136 analyzed transposition events in mouse liver were mapped within mouse RefSeq genes (**Fig. 5a**). When compared to a computer-simulated random integration profile with 26% of transposition events into RefSeq genes [27], these data revealed a similar or slightly reduced preference for SB-mediated integration into mouse genes. Notably, this frequency into genes is significantly lower when compared to murine leukemia virus (MLV, retroviral vectors) with 50.7% and human immunodeficiency virus derived vectors (lentiviral vectors) with 83.4% [39], [40]. Among the 31 transposition events that occurred in genes, 27 were mapped within intron sequences, and 4 were mapped in exons (**Fig. 5a**). The bias towards introns may be due to the larger overall size of introns when compared to exon sequences. Of the 105 integration sites in intergenic sequences, 8 (7.6%) were found near genes (±5 kb), while >70% were located 50 kb upstream or downstream of genes (**Fig. 5b**). This is in contrast to AAV vectors, for which over 38.3% of integrations were found near genes [41]. To evaluate the risk of neoplastic changes that could potentially be induced by the integrating vector system, those genes that were hit by transposition events or integration sites which were localized in close proximity of a gene (±5 kb), were checked in the Cancer Gene Expression Databases (CGED, http://lifesciencedb.jp/cged/) [31]. Although no neoplastic change was observed macroscopically in all treated animals, 10 transposition events from the AdV/SB hybrid-vectors were found to locate in or near cancer related genes (**Table S4**).

In addition to the 136 transposition events in the host genome, 27 transposon integration events (with 12 unique sites) were found to be localized within the HC-AdV genome used in this study (**Fig. 5c**). This indicates that the transposon was excised from the adenoviral vector and re-integrated into the HC-AdV genome at various TA-dinucleotides sites. Detailed analysis of insertion sites revealed, that they were located in the stuffer DNA contained in the HC-AdV genome (IgkMAR and alphoid repeat DNA) [3] and in the transgene expression cassette.

Multiple insertions were observed at two predominant sites of the HC-AdV genome. Four integration sites were located in the IgkMAR sequence derived from Mus musculus chromosome 6 (chr6∶70675584) and 8 integration sites were found within intron 1 of the human coagulation factor IX gene derived from the Homo sapiens chromosome X (chrX:138612987). The latter sequence was contained in the transgene expression cassette of the vector HC-AdV-TcFIX and located upstream of the cFIX cDNA sequence.

After performing the LAM-PCR method, insertion frequencies with respect to integration within or outside of RefSeq genes was analyzed and compared to a random dataset. As shown in **Fig. 5d** no significant change was observed with respect to integration into genes, intron and intergenic regions. Only transposition from the adenoviral vector into exons showed a slightly increased change in mouse f2 and m1 (fold change of >1). Compared to the random integration profile, there was also no significant change with respect to the distance of integration events upstream and downstream of genes (**Fig. 5d**).

### Genome Status of the Hybrid-vectors in Different Animals

The genome status and the genome copy numbers per cell of the transduced vectors may influence the safety profile of the AdV/SB hybrid-vector system. To further understand the molecular mechanism associated with this adenovirus/transposase hybrid-vector system, the genome copy numbers of each vector in liver genomic DNA of all treated animals were determined by quantitative real-time PCR after three CCl_4_ treatments. For analysis of cFIX and SB encoding sequence, the same primer pairs as for vector titration were used (**Fig. 1**). To quantify the total amount of adenoviral vector genomes we also used a primer pair specifically detecting the HC-AdV backbone. To normalize the different samples, the same amount of genomic DNA was analyzed by real-time PCR using mouse TBP (TATA box binding protein) for mouse samples and for dog samples B2M (Beta-2-microgloblin).

**Figure 6:**
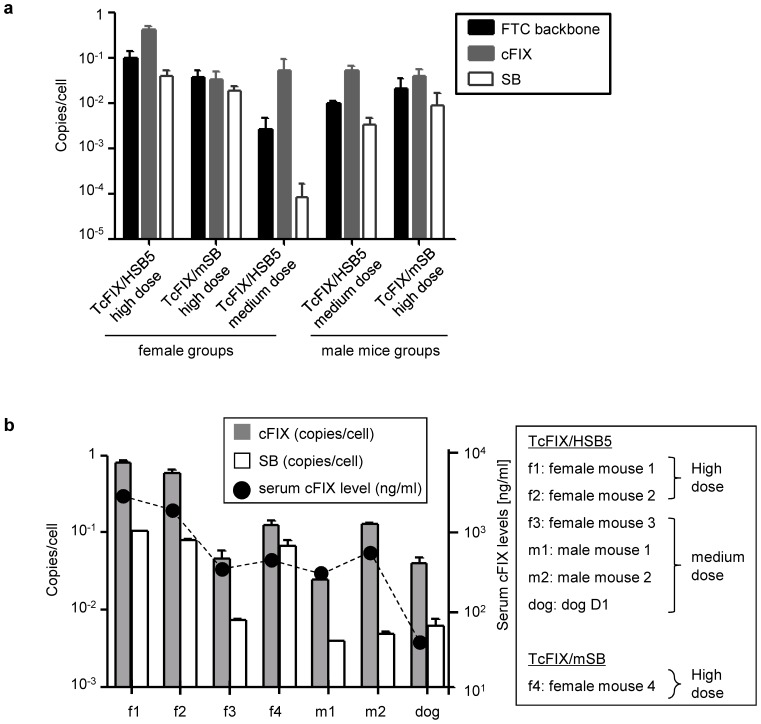
Vector genome copy numbers in liver after in vivo application. Liver genomic DNA was analyzed by quantitative real-time PCR using primers and probe detecting the HC-AdV vector backbone and primers detecting the cDNAs of the two main functional genes cFIX and SB. As internal control TBP for mouse and B2M for dog were used. (**a**) Analysis of the vector genome copy numbers in murine liver of female and male mice (n = 3 per group), with respect to sex, vector dose used and transposase type. (**b**) Vector genome copy numbers and serum cFIX levels of individual mice which were used for integration site analysis. Four female mice (f1–f4) and 2 male mice (m1, m2) were analyzed. As a comparison we also analyzed a sample derived from dog liver after hybrid-vector infusion [21]. Dog indicates dog D1 which received 9.4×10^11^ vps/kg (equals to 4.7×10^10^ TU/kg) [21]. Error bars indicate the standard deviations for each group.

In all treated animals which received HSB5, the cFIX gene copy numbers per cell were at least one log higher than vector genome copy numbers measured for the HSB5 transposase gene (**Fig. 6a**). However, this was different for mice which received the HC-AdV encoding mSB (**Fig. 6a**). Furthermore, for the PCR detecting the FTC backbone, copy numbers per cell were significantly lower compared to the cFIX copy numbers for mice which received the hyperactive HSB5 (**Fig. 6a**).

Next we analyzed mice which were used for integration site analysis (female mice f1–f3, male mice m1 and m2, mSB viral vector treated female mouse f4). Based on our previous study [21] and for direct comparison, we also analyzed one sample of a dog (dog 1) which was treated by the SB transposase hybrid-vector system showing long-term expression of cFIX for several years post-injection. In contrast to the results obtained from a semi-quantification PCR in our historical study [21], the SB transposase gene was detectable by real-time PCR in all animals ranging from 0.004 to 0.106 copies per cell. However, in all AdV/SB transposase hybrid-vectors treated animals except for the mouse f4 which received the inactive version of transposase (mSB), there was at average an 8-fold increase of cFIX gene copy numbers compared to the genome copy numbers measured for the SB transposase gene. It is of note that the two female mice f1 and f2, which received a high dose vector infusion (2×10^11^ TU/kg), highest genome copy numbers (0.811 and 0.581 copies/cell for the transposon-encoding sequence and 0.106 and 0.080 copies/cell for transposase-encoding sequence) and highest cFIX expression levels (4500 and 3100 ng/ml at the last time point measured) could be measured (**Fig. 6b**). Therefore, we could maintain almost one copy per cell of the transposon encoded transgene. In contrast, mice which received a medium vector dose (f3, m1 and m2, 4×10^10^ TU/kg) the genome copy numbers and transgene expression levels were lower (**Fig. 6b**) indicating that there is a correlation between vector dose and transgene expression. Notably, for all treated animals SB transposase sequences were still detectable, demonstrating that HC-AdV genomes encoding the transposase were still present in transduced liver. However, it remains to be analyzed whether SB transposase is still actively transcribed or whether the transgene expression cassette was silenced by epigenetic effects.

## Discussion

Herein, we analyzed efficacy and safety issues of our recently developed adenovirus-transposase hybrid-vector utilizing the hyperactive Sleeping Beauty transposase HSB5. This vector system was shown to result in stabilized transgene expression in mice even during rapid cell cycling and it was demonstrated that this hybrid-vector can mediate long-term phenotypic correction in a hemophilia B dog [21]. Herein, we carried out dose escalation studies and we evaluated genotoxicity which can potentially be caused by somatic integration of the transposon after transposition. We showed that the adenovirus-transposase hybrid-vector could mediate persistent transgene expression **(** **Fig. 2**), however, a vector dose as high as 2×10^11^ TU/kg led to lethal toxicity 5 to 14 day post-injection in a significant number of mice receiving various vector settings (**Table S2**). It is of note that healthy C57Bl/6 mice at 6–8 weeks of age were used for this study and that 1 day post-infusion ALT levels were increased while remaining in a normal range for all treated mice up to one week post-injection (20–80 U/l) (**Fig. 2d**). We observed fatalities in mice independent of the transgene encoded by the adenoviral vector in the high vector dose setting (2×10^11^ TUs/kg body weight, equals approximately 4.0×10^12^ VPs/kg body weight) (**Table S2**). Therefore, we hypothesize that expression of SB transposase and/or FLPe recombinase may not be the reason for the high mortality rate. Also supraphysiological cFIX expression levels can most likely be excluded as a reason for the high mortality rate, because to our knowledge there is no evidence in the literature that this may cause unwanted side effects in mice. However, adapted immune responses against the incoming human adenovirus capsid may have contributed at least in part to the observed toxicity. It is of notion that numerous previous studies showed that systemic administration of HC-AdVs led to dose-dependent acute toxicity. However, those toxicities were observed shortly after vector injection (<48 hrs) associated with activation of the innate inflammatory immune response [32], [42], [43]. Also, in one clinical trial using intravascular administration of HC-AdV at a dose of 4.3×10^11^ VPs/kg into a hemophilia A patient, transient inflammatory response with hematologic and liver abnormalities was observed [44]. It is of note, that the dose chosen in our study, even the high dose (2×10^11^ TU/kg, equals approximately 4.0×10^12^ VPs/kg) was tolerated in other studies. For instance, although associated with acute toxicity, 5.6×10^12^ VPs/kg of the gutless adenovirus HC-AdV-LacZ was a safe dose in baboons, and 7.5 × 10^12^ VPs/kg of the apoE expressing HC-AdV or 3×10^11^ particles per mouse of a VLDLR expressing HC-AdV were safe doses in mice [5], [45], [46]. Nevertheless, it can be concluded from this study that a dose of ≥2×10^11^ TU/kg of the HC-AdV/SB hybrid-vectors is above the maximum tolerable dose in mice and that a dose ≤8×10^9^ TU/kg is not sufficient for achieving stabilized and long-term transgene expression. Therefore, there seems to be a relatively narrow therapeutic window for the hybrid-vector system analyzed in the present study.

After performing LM-PCR and LAM-PCR to characterize transposition events, we identified 163 and 1164 integration site, respectively. We found that transposase-mediated integration events from the adenoviral vector genome displayed a nearly random integration profile with respect to integration into gene and non-gene regions (**Fig. 5a, d**). This finding was in concordance with results described in other studies using either a lentiviral-transposase hybrid-vector system **[28]**–**[30]** or a non-viral approach based on plasmids [27].

Interestingly, female mice (f1, f2 and f3) and male mice (m1 and m2) revealed a bias towards integration in the X-chromosome when using the LM-PCR method (47% for female mouse f1, 37% for female mouse f2, 13% for male mouse m1, 9.5% for male mouse m2) (**Fig. 3a** **and** **4c**), but the LAM-PCR showed no significantly increased bias towards the X-chromosome (**Figs. 3b** and **4d**). This result indicated that chromosomal distribution is influenced by the method used and the total number of identified integration sites. For instance, the LM-PCR method applied in the present study is strongly dependent on the restriction enzyme used for digestion of isolated genomic DNA, which may influence the distribution of recovered integration sites from host chromosomes. However, comparing the two methods for analysis of transposition events, one important advantage of the LM-PCR method is the fact, that this method can be used by any laboratory and not only by specialized groups. LAM-PCR on the other hand allows identifying a larger number of transposition events. It is of note, that our LM-PCR-based method was also used in a system utilizing PhiC31 integrase for somatic integration from the adenoviral vector [37] without finding a preference for the X-chromosome. In general it also needs to be considered that administration of CCl4 may influence the vector fate and the integration pattern. We believe, however, that transposition occurs within the first few days after vector injection and CCl4 injection was started several weeks after vector injection. Another issue which needs to be considered is the likelyhood that the remaining episomal adenoviral vectors may be inserted into the host genome when rapid cell cycling is induced by the liver toxin.

We identified 1164 transposition events in female and male mice referring to the mouse genome when applying the LAM-PCR method and it needs to be emphasized that the Illumina genome analyzer technology should have the potential to analyze even more integration sites. However, it is of note, that extra-chromosomal integration events into the delivered adenoviral vector referring to transposition events from the transposon-donor vector into the delivered episomal adenoviral vectors HC-AdV-TcFIX or HC-AdV-HSB5 were neglected when analyzing the LAM-PCR dataset. Since 27 of 163 transposition events (17%) were identified to be located in the delivered adenoviral vectors when using LM-PCR (**Fig. 5c**), even more extrachromosomal transposition events can be expected for the LAM-PCR approach. Extra-chromosomal target sites for SB transposase were also described in a plasmid-based study [27], in which the promoter region and the polyadenylation signal region were found to be a “hot spot” including 85% of all extra-chromosomal transposition events. The present study, however, showed no bias towards promoter or polyadenylation signal regions (**Fig. 5c**).

Copy number analysis showed detectable adenoviral vector genomes in all mouse groups (ranging from 10^−1^ to 10^−3^ copies per cell) (**Fig. 6a**), however, since the frequency of recombinant adenoviral vector genome integration is known to be rather low [8]–[10], we assume that most of these viral vector genomes remained episomal. Moreover, in the mouse groups which received the hyperactive transposase HSB5, the cFIX copy numbers detected were almost one log higher than that of the FTC backbone (**Fig. 6a**), indicating that the persistent cFIX expression is mainly associated with SB-mediated integration of the transgene and not the adenoviral genome persistence. Another concern from this study is that genomes of transposase encoding vector (HC-AdV-HSB5/mSB) were still detected in the hybrid-vectors administrated mice and the sample derived from one treated dog which is probability due to the persistence of the adenoviral vector genome (**Fig. 6**). It needs to be pointed out that duration of transposase expression was not analyzed in our study and that long-term exposure of liver cells to the transposase gene product may cause liver toxicity which may have a direct impact on cell growth. For instance, long-term high level SB transposase expression may cause secondary transposition, potentially resulting in an increased risk for neoplastic changes. However, the frequency of SB remobilization from mammalian cell chromosomes was described to be low (10^−4^ to 10^−6^ events per transfected cell) [47]–[49]. Another concern could be a direct impact of SB transposase expression on cell growth. For instance, Galla and colleagues observed a dose-dependent G2/M cell cycle arrest and induction of apoptosis caused by the over-expressed SB transposase [50]. Notably, in this study cytotoxicity was also observed in the absence of a co-delivered transposable element. In our study we used the PGK promoter to control expression of SB transposase (**Fig. 1a**). This PGK promoter is relatively weak and therefore, over-expression of SB transposase can be ruled out at the time. To actually address this question, further studies need to be performed analyzing transcription and expression levels of SB. Interestingly, a recent study examined SB transposase gene copy numbers after delivery of the SB system into primary human T cells. They also detected a relatively high copy number of SB transposase but neither the transposase mRNA nor protein was detectable [51]. Nevertheless, due to the high copy number of detectable transposase found in this and several other studies, SB transposase delivery needs to be carried out with caution. To avoid long-term expression of SB transposase, dose-controlled mRNA delivery or delivery of an expression cassette for which the transposase gene is expressed under the control of an inducible promoter could be performed [50]–[52].

In summary, our results demonstrated that the AdV/SB hybrid-vector system can facilitate long-term stable transgene expression in a vector dose-dependent manner, but the vector dose need to be strictly limited to non-toxic level (must be below 2×10^11^ TU/kg). Importantly, this report describes for the first time a genome-wide analysis of transposition events after application of the AdV/SB hybrid-vector system *in vivo*. The relatively lower percentage of transposition events identified in genes is encouraging with respect to genotoxicity.

## Supporting Information

Figure S1 **Schematic outline of a ligation-mediated PCR (LM-PCR) method to determine integration sites.** (**a**) Genomic DNA digest: highly purified genomic DNA was digested with four blunt-ended restriction enzyme nucleases: *Ssp*I, *Pvu*II, *Stu*I, *Eco*RV. (**b**) Linker ligation: linker from the BD GenomeWalker™ kit was ligated to the blunt ends of the genomic DNA fragments creating four libraries. (**c**) Two-step PCR: the first PCR was performed with a gene-specific primer (GSP1) binding to the transposon flanked sequence IR, and an adaptor specific primer (ASP1) which specifically recognized the 5′ end of the adaptor-ligated genomic fragments. The second PCR was a nested PCR performed with primers binding to sequences located within the first PCR product (GSP2 and ASP2). (**d**) Subcloning and sequencing of PCR products containing the chromosomal DNA flanking the integrated transposon: PCR products generated by the nested PCR were subcloned into the pCR-blunt II-TOPO vector (3.5 kb). After screening of clones by EcoRI digestion, the genomic DNA-transposon interface sequence was sequenced for each clone and chromosomal location of the genomic DNA identified.
(TIF)

Figure S2 **Establishment of the ligation-mediated PCR (LM-PCR) method to determine integration sites based on the GenomeWalker Kit (BD).** (**a**) Methodical set-up for evaluation of SB-mediated transposition from plasmids in cultured mammalian cells. Hela cells were co-transfected with the donor plasmid pTnori containing an expression cassette for neomycin and the plasmid pCMV-HSB5 encoding the hyperactive Sleeping Beauty transposase HSB5 or pCMV-mSB containing a non-functional version of the transposase. After transfection, cells were cultured for 14 days under selection with G418 for neomycin resistant cells. DNA isolated from grown cell colony pools were used for establishment of the LM-PCR method for analysis of integration sites. IR, transposon inverted repeats; SV40, simian virus promoter; neo, neomycin-phosphotransferase gene; Tn5: bacterial promoter; ori: bacterial ori; CMV, cytomegalovirus promoter; HSB5: hyperactive Sleeping Beauty transposase HSB5; mSB: mutated, non-functional Sleeping Beauty transposase. (**b**) Quality of genomic DNA extracted from transduced cells. The size of genomic DNA was analyzed by loading 100 ng of genomic DNA on a 0.6% agarose/EtBr gel. For optimal processing, genomic DNA should be bigger than 50 kb and no or minimal smearing should be visible indicating low degree of degradation. M, peqGOLD 1 kb DNA-Ladder (peqlab); Lanes 1–3, genomic DNA isolated from three independent dishes with pools of Hela cells containing somatically integrated transgenic DNA. (**c**) Control for sufficient digestion of genomic DNA with restriction enzymes *Ssp*I, *Pvu*II, *Stu*I, *Eco*RV. The degree of digestion of the genomic DNA for generation of respective libraries was controlled by loading 100 ng on a 0.6% agarose/EtBr gel, a smooth smear in each lane shows that the genomic DNA can be completely digested by the restriction enzymes. (**d**) Evaluation of the primary PCR product: 20 µl and 5 µl of the primary PCR products (total volume: 50 µl) were separated on a 1.5% agarose/EtBr gel. For a successful PCR, a multiple banding or a smearing pattern could be observed with product sizes ranging from 500 bp to 5 kb except for the negative control (H_2_O). Depicted arrows indicate bands visualizing distinct PCR products. (**e**) Evaluation of secondary PCR products (nested PCR): 5 µl of the secondary PCR products were analyzed on a 1.5% agarose/EtBr gel and respective products should be present as prominent bands within the range from 200 bp to 6 kb. (**f**) Visualization of clones of the integration site library. Size differences of plasmids containing the subcloned PCR products within the pCR-blunt II-TOPO vector indicate different integration events. (**g**) Screening of integration site library for unique integration events by restriction enzyme digest. A EcoRI restriction digest is performed to excise subcloned DNA fragments. This allows identification of bands with an unique size visualized on an agarose gel (valid PCR products should be bigger than 0.2 kb to enable sequencing and subsequent identification).
(TIF)

Figure S3 **Features of the analyzed transposition events using linker-mediated PCR (LM-PCR). (a)** Criteria and analysis of DNA sequences and transposition events determined by the LM-PCR method. Within the plasmids generated by subcloning of PCR-amplified genomic fragments, the genomic DNA sequences are flanked by the vector sequence and the IR at one side and the linker sequence and the vector sequence on the other side. The IR sequence consists of 34 base pairs containing the sequence of the nested primer GSP2 and the rest of the flanking part of the IR. The sequence next to the end of the IR end regularly begins with a TA-dinucleotide sequence and resembles the genomic location of the integration site. The linker consisting of 36 bps contains the sequence of the linker restricted by the nested primer ASP2. Furthermore, the genomic sequence and the linker is separated by the part of the restriction enzyme (RE) site remaining from the digestion step during the generation of the genomic library. SP6/T7: sequencing primers used for identification of inserts incorporated into the pCR-blunt II-TOPO vector; IR: inverted repeat; TA: dinucleotide sequence (genomic target site for the SB transposase). (**b**) Analysis of sequenced Sleeping Beauty mediated transposition sites within the genome of Hela cells. For verification of a SB-mediated integration site, the sequence of the nested primer (underlined), the sequence at the end of the inverted-repeat (IR; depicted in italic letters) and the TA dinucleotide target site (central shaded box) have to be within the determined sequence. * non-specific PCR product; # random integration event; § background (sequence derived from the donor-vector containing the transposon). (**c**) Screening of integration sites based on restriction enzyme analysis after PCR amplification of integration sites utilizing the linker-based method and subsequent subcloning in the TOPO cloning vector. *Eco*RI restriction enzyme digests of subcloned PCR products derived from HSB5 mouse f1 (left side) and mSB mouse f4 (right side) are shown. For subsequent sequencing of PCR products subcloned fragments smaller than 0.2 kb were excluded.
(TIF)

Table S1 **Oligonucleotides used in this study.**
(DOC)

Table S2 **Fate of all HC-AdV injected mice (summary of survival rate).**
(DOC)

Table S3 **Summary of the recovery rate of integration events based on the ligation-mediated PCR (LM-PCR) method.**
(DOC)

Table S4 **Transposon insertions in or near cancer related genes using LM-PCR.**
(DOC)
