# Correction: Integration Profile and Safety of an Adenovirus Hybrid-Vector Utilizing Hyperactive Sleeping Beauty Transposase for Somatic Integration

**DOI:** 10.1371/journal.pone.0108836

**Published:** 2014-09-18

**Authors:** The PLOS ONE Staff

“E-rare project Transposmart” was omitted as a funder for this article. Please refer to the complete funding statement below:

This work was supported in part by DFG grant SPP 1230 (A.E.), the E-rare project Transposmart (A.E. and Z.I.), the Wilhelm Sander-Foundation (A.E.), EU Framework Programme 7 (Persistent Transgenesis) (A.E), and the Friedrich-Baur-Foundation (A.E.). W.Z. was supported by a fellowship from the Chinese Scholarship Council (CSC) in cooperation with Northwest A&F University, Yangling, China. The funders had no role in study design, data collection and analysis, decision to publish, or preparation of the manuscript.
