# Correction: Integration Profile and Safety of an Adenovirus Hybrid-Vector Utilizing Hyperactive Sleeping Beauty Transposase for Somatic Integration

**DOI:** 10.1371/journal.pone.0228902

**Published:** 2020-02-04

**Authors:** Wenli Zhang, Martin Muck-Hausl, Jichang Wang, Chuanbo Sun, Maren Gebbing, Csaba Miskey, Zoltan Ivics, Zsuzsanna Izsvak, Anja Ehrhardt

The following information is missing from the Funding statement: Dr. Zsuzsanna Izsvák was funded by European Research Council-2011-ADG-TRANSPOSOstress– 294742. Additional information was added to the Funding statement in the Correction published in 2013 [2]. The complete, correct Funding statement is as follows: This work was supported in part by DFG grant SPP 1230 (A.E.), the E-rare project Transposmart (A.E. and Z.I.), the Wilhelm Sander-Foundation (A.E.), EU Framework Programme 7 (Persistent Transgenesis) (A.E), and the Friedrich-Baur-Foundation (A.E.). W.Z. was supported by a fellowship from the Chinese Scholarship Council (CSC) in cooperation with Northwest A&F University, Yangling, China. Dr. Zsuzsanna Izsvák was funded by European Research Council-2011-ADG-TRANSPOSOstress– 294742. The funders had no role in study design, data collection and analysis, decision to publish, or preparation of the manuscript.
