# Supplementary figures and images for: Integration Profile and Safety of an Adenovirus Hybrid-Vector Utilizing Hyperactive Sleeping Beauty Transposase for Somatic Integration
Source: PLoS One. 2013 Oct 4;8(10):e75344. doi: 10.1371/journal.pone.0075344 (PMC3790794; doi:10.1371/journal.pone.0075344)

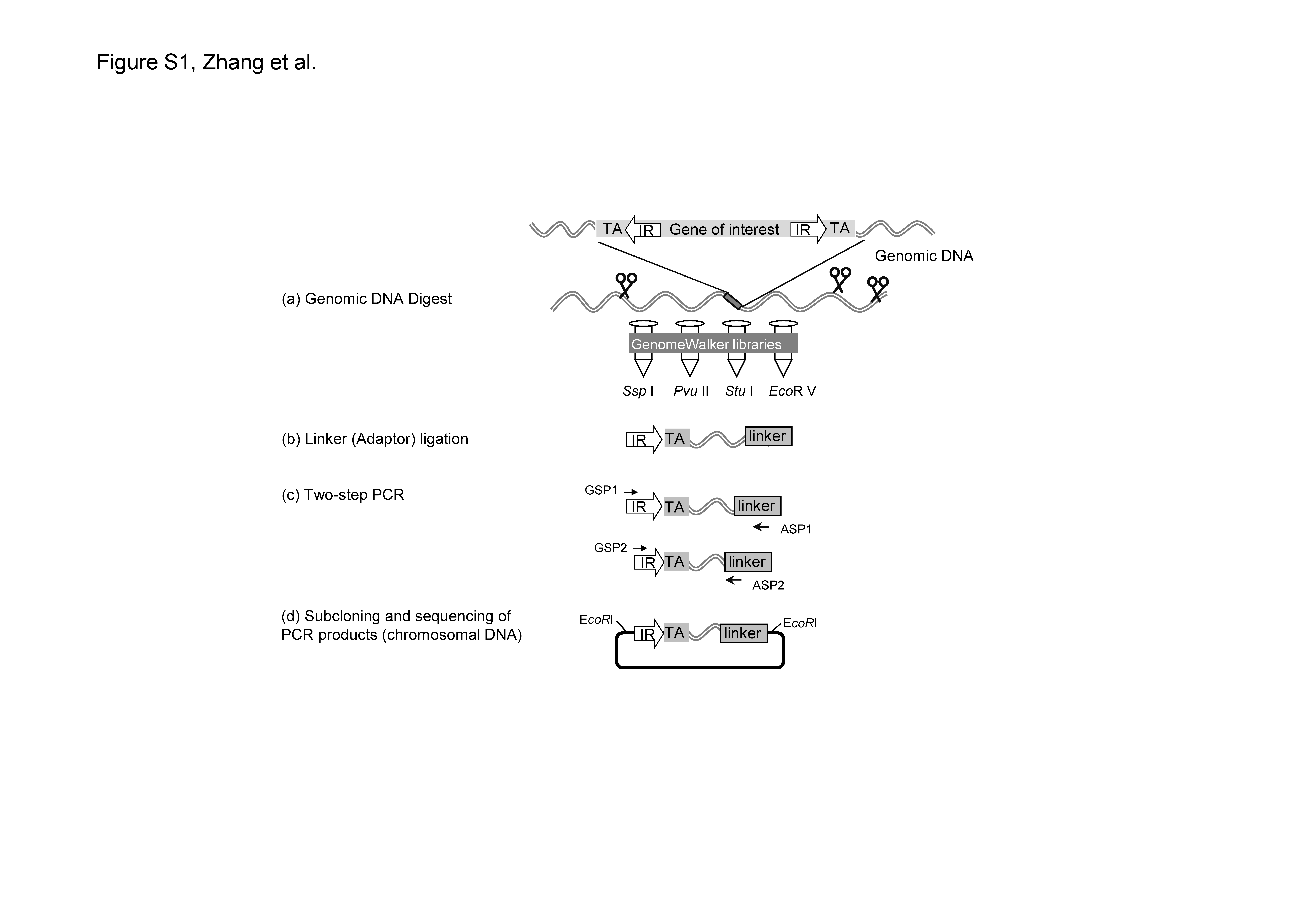

Supplement: Figure S1 — Schematic outline of a ligation-mediated PCR (LM-PCR) method to determine integration sites. (a) Genomic DNA digest: highly purified genomic DNA was digested with four blunt-ended restriction enzyme nucleases: SspI, PvuII, StuI, EcoRV. (b) Linker ligation: linker from the BD GenomeWalker™ kit was ligated to the blunt ends of the genomic DNA fragments creating four libraries. (c) Two-step PCR: the first PCR was performed with a gene-specific primer (GSP1) binding to the transposon flanked sequence IR, and an adaptor specific primer (ASP1) which specifically recognized the 5′ end of the adaptor-ligated genomic fragments. The second PCR was a nested PCR performed with primers binding to sequences located within the first PCR product (GSP2 and ASP2). (d) Subcloning and sequencing of PCR products containing the chromosomal DNA flanking the integrated transposon: PCR products generated by the nested PCR were subcloned into the pCR-blunt II-TOPO vector (3.5 kb). After screening of clones by EcoRI digestion, the genomic DNA-transposon interface sequence was sequenced for each clone and chromosomal location of the genomic DNA identified. (TIF) [file pone.0075344.s001.tif]

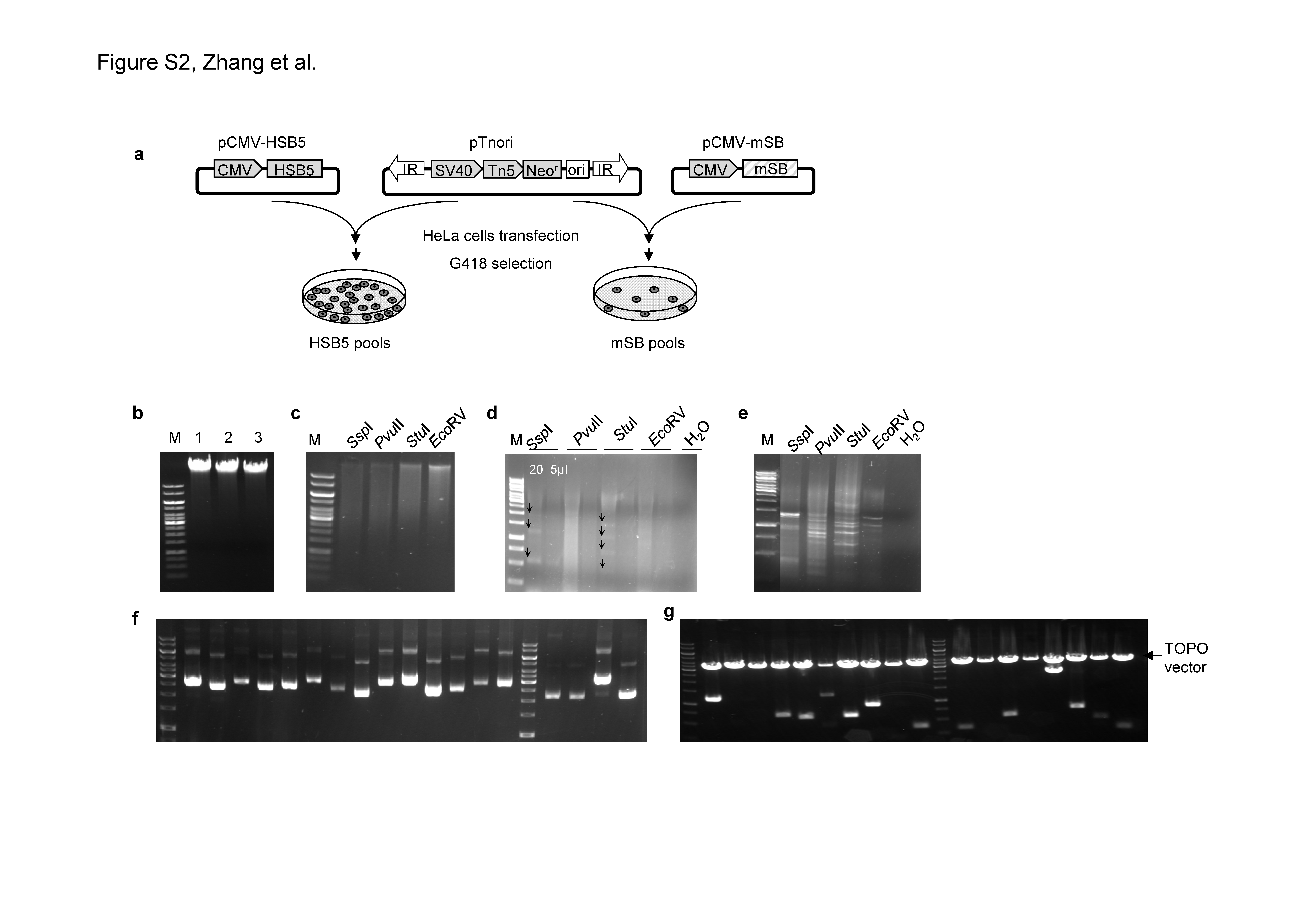

Supplement: Figure S2 — Establishment of the ligation-mediated PCR (LM-PCR) method to determine integration sites based on the GenomeWalker Kit (BD). (a) Methodical set-up for evaluation of SB-mediated transposition from plasmids in cultured mammalian cells. Hela cells were co-transfected with the donor plasmid pTnori containing an expression cassette for neomycin and the plasmid pCMV-HSB5 encoding the hyperactive Sleeping Beauty transposase HSB5 or pCMV-mSB containing a non-functional version of the transposase. After transfection, cells were cultured for 14 days under selection with G418 for neomycin resistant cells. DNA isolated from grown cell colony pools were used for establishment of the LM-PCR method for analysis of integration sites. IR, transposon inverted repeats; SV40, simian virus promoter; neo, neomycin-phosphotransferase gene; Tn5: bacterial promoter; ori: bacterial ori; CMV, cytomegalovirus promoter; HSB5: hyperactive Sleeping Beauty transposase HSB5; mSB: mutated, non-functional Sleeping Beauty transposase. (b) Quality of genomic DNA extracted from transduced cells. The size of genomic DNA was analyzed by loading 100 ng of genomic DNA on a 0.6% agarose/EtBr gel. For optimal processing, genomic DNA should be bigger than 50 kb and no or minimal smearing should be visible indicating low degree of degradation. M, peqGOLD 1 kb DNA-Ladder (peqlab); Lanes 1–3, genomic DNA isolated from three independent dishes with pools of Hela cells containing somatically integrated transgenic DNA. (c) Control for sufficient digestion of genomic DNA with restriction enzymes SspI, PvuII, StuI, EcoRV. The degree of digestion of the genomic DNA for generation of respective libraries was controlled by loading 100 ng on a 0.6% agarose/EtBr gel, a smooth smear in each lane shows that the genomic DNA can be completely digested by the restriction enzymes. (d) Evaluation of the primary PCR product: 20 µl and 5 µl of the primary PCR products (total volume: 50 µl) were separated on a 1.5% agarose/EtBr gel [file pone.0075344.s002.tif]

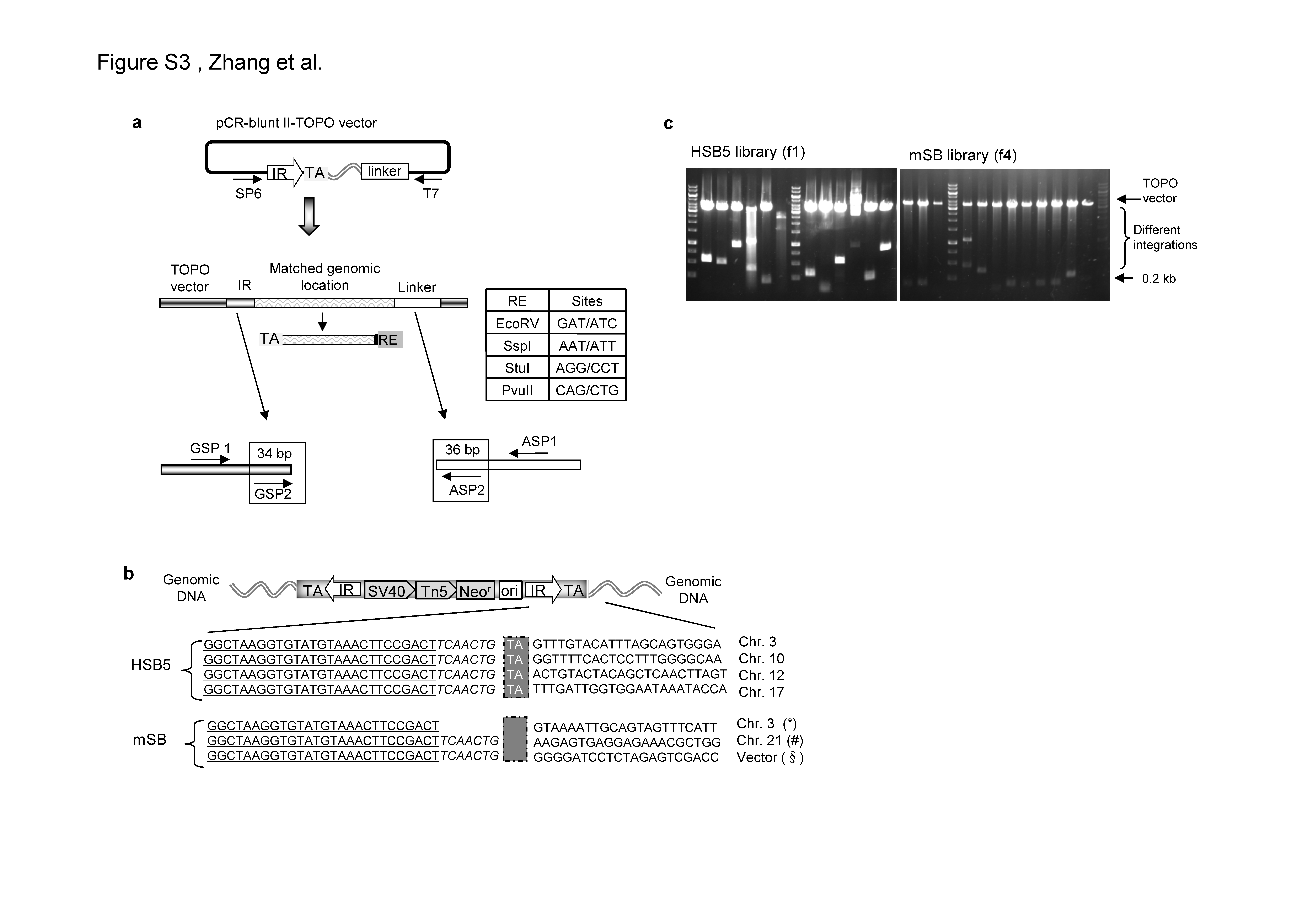

Supplement: Figure S3 — Features of the analyzed transposition events using linker-mediated PCR (LM-PCR). (a) Criteria and analysis of DNA sequences and transposition events determined by the LM-PCR method. Within the plasmids generated by subcloning of PCR-amplified genomic fragments, the genomic DNA sequences are flanked by the vector sequence and the IR at one side and the linker sequence and the vector sequence on the other side. The IR sequence consists of 34 base pairs containing the sequence of the nested primer GSP2 and the rest of the flanking part of the IR. The sequence next to the end of the IR end regularly begins with a TA-dinucleotide sequence and resembles the genomic location of the integration site. The linker consisting of 36 bps contains the sequence of the linker restricted by the nested primer ASP2. Furthermore, the genomic sequence and the linker is separated by the part of the restriction enzyme (RE) site remaining from the digestion step during the generation of the genomic library. SP6/T7: sequencing primers used for identification of inserts incorporated into the pCR-blunt II-TOPO vector; IR: inverted repeat; TA: dinucleotide sequence (genomic target site for the SB transposase). (b) Analysis of sequenced Sleeping Beauty mediated transposition sites within the genome of Hela cells. For verification of a SB-mediated integration site, the sequence of the nested primer (underlined), the sequence at the end of the inverted-repeat (IR; depicted in italic letters) and the TA dinucleotide target site (central shaded box) have to be within the determined sequence. * non-specific PCR product; # random integration event; § background (sequence derived from the donor-vector containing the transposon). (c) Screening of integration sites based on restriction enzyme analysis after PCR amplification of integration sites utilizing the linker-based method and subsequent subcloning in the TOPO cloning vector. EcoRI restriction enzyme digests of subcloned PCR products derived from [file pone.0075344.s003.tif]
