# Supplementary material for: Integration Profile and Safety of an Adenovirus Hybrid-Vector Utilizing Hyperactive Sleeping Beauty Transposase for Somatic Integration
Source: PLoS One. 2013 Oct 4;8(10):e75344. doi: 10.1371/journal.pone.0075344 (PMC3790794; doi:10.1371/journal.pone.0075344)
Supplement: Table S1 — Oligonucleotides used in this study. (DOC) [file pone.0075344.s004.doc]

**Table S1. Oligonucleotides used in this study.**

| **Target/Name** | **Orientation** | **Sequence (5’ to 3’)** | **Reference** |
| --- | --- | --- | --- |
| ASP1 | Forward | GTAATACGACTCACTATAGGGC | BD |
| ASP2 | Reverse | ACTATAGGGCACGCGTGGT |
| Dog B2M-F | Forward | GGATGAGTTTAGCTGCCGTG |  |
| Dog B2M-R | Reverse | TATCTGAGCACAGGCACAGC |
| E1-F | Forward | GGGTGAGGAGTTTGTGTTAGATTATG | [47] |
| E1-R | Reverse | TCCTCCGGTGATAATGACAAGA |
| E1-specific probe |  | [6-FAM] AGC ACC CCG GGC ACG GTTG [TAMAR] |
| FTC-backbone f | Forward | TGC GGA GAA AGA GGT AAT GAA |  |
| FTC-backbone r | Reverse | TAC CGA GCT CGA ATT GAT CC |
| FTC-backbone probe |  | [6-FAM] CGGTCCGACGTCCTAGG  CGTTTTATTAT [TAMAR] |
| GSP1 | Forward | CCTTAAGACAGGGAATCTTTACTCGGA |  |
| GSP2 | Reverse | GGCTAAGGTGTATGTAAACTTCCGACT |
| hAAT-cFIX f | Forward | CTG ACC TGG GAC AGT GAA TGA T |
| hAAT-cFIX r | Reverse | GCC TGG TGA TTC TGC CAT GAT |
| L-IR | Forward | GGCAAGTCAGTTAGGACATCT |  |
| L3-F | Forward | GAG TTG GCA CCC CTA TTC GA | [47] |
| L3-R | Reverse | ATG CCA CAT CCG TTG ACT TG |
| L3-specific probe |  | [6-FAM] CCA CCC GTG TGT ACC TGG TGG ACA [TAMAR] |
| Linker for genome walker PCR |  | GTAATACGACTCACTATAGGGCACGCGTGGTCGACGGCCCGGGCTGGT–3'  3'–H2N-CCCGACCA-PO4–5' | BD |
| Mouse TBP f | Forward | CCCCACAACTCTTCCATTCT |  |
| Mouse TBP r | Reverse | GCAGGAGTGATAGGGGTCAT |
| R-IR | Forward | CCTTAAGACAGGGAATCTTTACTC |  |
| SB-L | Forward | GGTGGCAGCATCATGTTGTG |  |
| SB-R | Reverse | CCTTCCTCATGATGCCATCTATT |

BD, BD GenomeWalker^TM^ Kit from BD Biosciences
