# Supplementary material for: Integration Profile and Safety of an Adenovirus Hybrid-Vector Utilizing Hyperactive Sleeping Beauty Transposase for Somatic Integration
Source: PLoS One. 2013 Oct 4;8(10):e75344. doi: 10.1371/journal.pone.0075344 (PMC3790794; doi:10.1371/journal.pone.0075344)
Supplement: Table S2 — Fate of all HC-AdV injected mice (summary of survival rate). (DOC) [file pone.0075344.s005.doc]

**Table S2. Fate of all HC-AdV injected mice (summary of survival rate).**

| **Group** | **Viral vector injection** | | **Fate of all injected mice** | | | | |
| --- | --- | --- | --- | --- | --- | --- | --- |
| Mouse ID | Vector Type | Dose (TU/mouse) | Total (n=) | Survived (n=) | Died (n=) | Survival rate (%) |  |
| **Female mice** | |  |  |  |  |  |  |
| 0808-3010 | HC-AdV-TcFIX/HSB5 | 4.0 x 10^9^ | 3 | 1 | 2 (5 dpi) | **33** |  |
| 1905-0409 | HC-AdV-TcFIX/HSB5 | 4.0 x 10^9^ | 5 | 3 | 2 (7 dpi) | **60** |  |
| 2302-3007 | HC-AdV-TcFIX/HSB5 | 4.0 x 10^9^ | 5 | 1 | 2 (8 dpi)  2 (12 dpi) | **20** |  |
| 0808-3010 | HC-AdV-TcFIX/mSB | 4.0 x 10^9^ | 3 | 3 | 0 | **100** |  |
| 1905-0409 | HC-AdV-TcFIX/mSB | 4.0 x 10^9^ | 5 | 4 | 1 (7 dpi) | **80** |  |
| 2302-3007 | HC-AdV-TcFIX/mSB | 4.0 x 10^9^ | 5 | 5 | 0 | **100** |  |
| 2302-1508 | HC-AdV-TcFIX/Luc | 4.0 x 10^9^ | 5 | 2 | 1 (9 dpi), 1 (16 dpi)  1 (24 dpi) | **40** |  |
| 3003-2508 | HC-AdV-TcFIX/Luc | 8.0 x 10^8^ | 10 | 9 | 1 (after CCl_4_) | **90** |  |
| 2302-3007 | HC-AdV-TcFIX/HSB5 | 8.0 x 10^8^ | 5 | 4 | 1 (after CCl_4_) | **80** |  |
| 3003-2508 | HC-AdV-TcFIX/HSB5 | 1.6 x 10^8^ | 5 | 3 | 2 (after CCl_4_) | **60** |  |
| **Male mice** | |  |  |  |  |  |  |
| 2302-3007 | HC-AdV-TcFIX/HSB5 | 4.0 x 10^9^ | 5 | 2 | 1 (9 dpi), 1 (11 dpi)  1 (12 dpi) | **40** |  |
| 2302-3007 | HC-AdV-TcFIX/mSB | 4.0 x 10^9^ | 5 | 5 | 0 | **100** |  |
| 2302-3007 | HC-AdV-TcFIX/Luc | 4.0 x 10^9^ | 5 | 1 | 1 (9 dpi),1 (11 dpi)  1 (15 dpi),1 (30 dpi) | **20** |  |
| 2302-3007 | HC-AdV-TcFIX/HSB5 | 8.0 x 10^8^ | 5 | 5 | 0 | **100** |  |
| **total** |  |  | **65** | **44*** | **21*** | **70** |  |

*4 mice died due to the CCl4 injection; dpi, days post-injection; If CCl_4_ treatment was associated with fatalities in mice, the number of the survival rate was underlined; High dose: 4.0 x 10^9^ TU/mouse; Middle dose: 8.0 x 10^8^ TU/mouse; Low dose: 1.6 x 10^8^ TU/mouse.
