# Supplementary material for: Integration Profile and Safety of an Adenovirus Hybrid-Vector Utilizing Hyperactive Sleeping Beauty Transposase for Somatic Integration
Source: PLoS One. 2013 Oct 4;8(10):e75344. doi: 10.1371/journal.pone.0075344 (PMC3790794; doi:10.1371/journal.pone.0075344)
Supplement: Table S3 — Summary of the recovery rate of integration events based on the ligation-mediated PCR (LM-PCR) method. (DOC) [file pone.0075344.s006.doc]

**Table S3. Summary of the recovery rate of integration events based on the ligation-mediated PCR (LM-PCR) method.**

|  | **F1** | **F2** | **F3** | **M1** | **M2** | **mSB** | **total** |
| --- | --- | --- | --- | --- | --- | --- | --- |
| Colonies picked | 65 | 70 | 64 | 80 | 70 | 36 | 385 |
| Clones identified (by diagnostic restriction enzyme digest) | 47 | 50 | 50 | 49 | 43 | 16 | 255 |
| Cloning vectors identified | 2 | 6 | 3 | 7 | 7 | 9 | 34 |
| Non-specific PCR products (without intact IR or TA) | 4 | 3 | 9 | 7 | 5 | 7 | 35 |
| Failed sequence | 1 | 6 | 19 | 12 | 7 | 0 | 45 |
| **Integration events** | 40 | 35 | 19 | 23 | 24 | 0 | 141 |
| recovery rate (% of real integration events in all the picked colonies) | 62 | 50 | 30 | 29 | 34 | 0 | 20 |
