# Supplementary material for: Integration Profile and Safety of an Adenovirus Hybrid-Vector Utilizing Hyperactive Sleeping Beauty Transposase for Somatic Integration
Source: PLoS One. 2013 Oct 4;8(10):e75344. doi: 10.1371/journal.pone.0075344 (PMC3790794; doi:10.1371/journal.pone.0075344)
Supplement: Table S4 — Transposon insertions in or near cancer related genes using LM-PCR. (DOC) [file pone.0075344.s007.doc]

**Table S4. Transposon insertions in or near cancer related genes using LM-PCR.**

| **mouse** | **chromosomal location** | **Band** | **gene symbol & name** | **cancer type** |
| --- | --- | --- | --- | --- |
|  | chr5: 92880283 | 5 E2 | SCARB2: scavenger receptor class B, member 2 | gastric (GC) |
| **f1** | chrx: 49859131 | XA5 | GPC3: glypican 3 | hepatocellular (HCC) |
|  | chrx: 13341506 | XA1.1 | CASK: calcium/calmodulin-dependent serine protein kinase | hepatocellular (HCC) |
|  | chr2: 154999131 | 2H1 | ITCH: itchy, E3 ubiquitin protein ligase | hepatocellular (HCC) |
| **f2** | chrx: 138774886 | XF2 | ACSL4: acyl-CoA synthetase long-chain family member 4 | breast (BC) |
|  | chr19: 43843935 | 19C3 | 1.1kb up to CUTC: cutC copper transporter homolog | gastric (GC) |
|  | chr1: 37535572 | 1B | MGAT4A: mannoside acetylglucosaminyltransferase 4, isoenzyme A | thyroid (TC) |
| **m1** | chr11: 21917357 | 11A3.1 | EHBP1: EH domain binding protein 1 | gastric (GC) |
|  | chrx: 20425116 | XA1.3 | 1.99kb down after ARAF: v-raf murine sarcoma 3611 viral oncogene homolog | colorectal (CC) |
| **m2** | chr5: 36106310 | 5B3 | ABLIM2: actin-binding LIM protein 2 | gastric (GC) |
